# Conservation status and regional habitat priorities for the Orinoco crocodile: Past, present, and future

**DOI:** 10.1371/journal.pone.0172439

**Published:** 2017-02-24

**Authors:** Sergio A. Balaguera-Reina, Ariel S. Espinosa-Blanco, Mónica A. Morales-Betancourt, Andrés E. Seijas, Carlos A. Lasso, Rafael Antelo, Llewellyn D. Densmore

**Affiliations:** 1 Department of Biological Sciences, Texas Tech University, Lubbock, Texas, United States of America; 2 Laboratorio de Ecología y Genética de Poblaciones, Centro de Ecología, Instituto Venezolano de Investigaciones Científicas (IVIC), Caracas, Venezuela; 3 Grupo de Estudios Ornitológicos y Fauna Silvestre, GEO-UPTC, Universidad Pedagógica y Tecnológica de Colombia, UPTC, Tunja, Colombia; 4 Instituto de Investigación de Recursos Biológicos Alexander von Humboldt, Bogotá, D.C., Colombia; 5 Universidad Nacional Experimental de los Llanos Occidentales “Ezequiel Zamora” UNELLEZ, Guanare, Venezuela; 6 Fundación Palmarito Casanare, Bogotá, Colombia, Dpto. Biodiversidad y Biología Evolutiva, Museo Nacional de Ciencias Naturales, CSIC, Madrid, Spain; Sichuan University, CHINA

## Abstract

Conservation of large predator species has historically been a challenge because they often overlap in resource utilization with humans; furthermore, there is a general lack of in-depth knowledge of their ecology and natural history. We assessed the conservation status of the Orinoco crocodile (*Crocodylus intermedius*), defining regional habitat priorities/crocodile conservation units (RHP/CCU) and regional research priorities (RRP) for this species. We also estimated a species distribution model (SDM) to define current suitable areas where the species might inhabit and/or that might be successfully colonized. The SDM area obtained with a suitable habitat probability ≥ 0.5 was 23,621 km^2^. Out of 2,562 km^2^ are included within protected areas in both Colombia (1,643 km^2^) and Venezuela (919 km^2^), which represents only 10.8% of *C*. *intermedius*’ potential range. Areas such as Laguna de Chigüichigüe (flood plain lagoon) exhibited an increase in population abundance. In contrast, localities such as the Cojedes and Manapire Rivers reported a significant reduction in relative abundance values. In Colombia, disparity in previous survey methods prevented accurate estimation of population trends. Only one study in this country described an increase over a 13 years span in the Ele, Lipa, and Cravo Norte River populations based on nest surveys. We defined 34 critical areas (16 in Colombia, 17 in Venezuela, and one covering both countries) where we need to preserve/research/monitor and/or generate management actions, 10 RHP/CCU (six from Venezuela and four from Colombia) and 24 RRP (11 from Venezuela, 12 from Colombia, and one in both countries). Caño Guaritico (Creek) and the Capanaparo River in Venezuela and the Ele, Lipa, Cravo Norte River System and the Guayabero River in Colombia were defined as areas with the most optimal conditions for long-term preservation and maintenance of *C*. *intermedius* populations. We conclude that the conservation status of this species is still critical, which implies the necessity to increase efforts to recover the species, especially in Colombia, to guarantee its survival as a structural and functional component of the ecosystems it inhabits.

## Introduction

Large predator conservation has historically been a significant challenge for biologists because these animals compete (either directly or indirectly) with humans for space and resources, and in many cases, they are considered a direct threat to human safety [1]. It also has been difficult due to the absence of thorough knowledge about the ecology and natural history of these predators [2]. The Orinoco crocodile (*Crocodylus intermedius*) is an example of such a species. *C*. *intermedius* is one of the most seriously threatened Neotropical crocodylians due to historical over-exploitation by hunters and poachers, restricted distribution, and habitat loss [3]. It is currently listed as critically endangered (CR) under the International Union for the Conservation of Nature Red List (IUCN Red List) and included in the Appendix I of the Convention on International Trade in Endangered Species of wild fauna and flora (CITES) [4]. Regionally, *C*. *intermedius* is catalogued as CR in Colombia [5] and endangered (EN) in Venezuela [6].

The Orinoco crocodile is also one of the largest crocodylians in the Neotropics inhabiting a restricted area in the middle and lower basins of the Orinoco River and its tributaries in Venezuela and Colombia [7]. As many crocodylians do, *C*. *intermedius* inhabits a wide variety of aquatic-land habitats, including rivers in tropical evergreen forests and piedmont streams in the foothills of the Andean mountains [8], however to date the highest population numbers for the species have been recorded from seasonal rivers (flood plain environments) in savanna ecosystems [3].

Despite the number of population ecology studies and restocking programs developed during the last two decades (mainly in Venezuela), reliable information about the status of populations in both Venezuela [9] and Colombia [10] is lacking. Recent studies suggest that populations in areas such as the Cojedes River System and Capanaparo River, which were long considered healthy, are declining [9,11]. On the other hand, technical and integrative analyses of Orinoco crocodiles (i.e., covering all its range and including habitat status, population and spatial ecology, and human-crocodile interactions) are lacking, limiting the capacity to define regional conservation priorities as well as information gaps and research priorities.

Colombia currently has 32% of its territory (including marine and land areas) under local or regional categories of protection, compared to 57% of Venezuelan territory that is currently protected [12]. Nevertheless, the lack of current analyses regarding the overall distribution of *C*. *intermedius* has prevented estimation of how much suitable habitat for this species is included in these protected areas, as well as whether or not the conservation measures in place are bringing any benefit to the overall protection of Orinoco crocodile populations.

Based upon current technical knowledge, regional habitat priorities (RHP) are aimed at classifying areas having high, medium, and low levels of conservation of populations, habitats, and management actions, respectively. This classification purports to increase allocation of resources in an optimal manner in terms of both research effort and management strategy, helping decision-makers to support initiatives. RHP have been defined in other species, such as *Crocodylus acutus* (the American crocodile) [13] with reasonable success in several countries (e.g., Panama), where they have been included in management actions [14]. For *C*. *intermedius*, several pilot approaches of RHP have been done in Venezuelan [15] and Colombian populations [16]. However, lack of implementation and follow-up discussions, as well as habitat transformation during the past 20 years, highlight the necessity to update these analyses.

Although there are current management plans for the Orinoco crocodile in both countries [3,17–19] and priorities were defined and established almost three decades ago, *C*. *intermedius* is still considered a threatened species. Thus, we assessed the conservation status of the Orinoco crocodile based on a thorough data revision, defining regional habitat priorities (RHP, also defined by some colleagues [13] as crocodile conservation units-CCU) and regional research priorities (RRP) for the species throughout its range, analyzing the past, present, and future conservation of the species. We also used a species distribution model (SDM) based only on high-quality and verifiable geolocations to define the current suitable areas where the species can potentially inhabit and/or areas *susceptible* to colonization, to determine its current habitat conservation status.

## Methods

We carried out a thorough data review of the spatial ecology (localities and geolocations), habitat status, and population ecology (relative abundances, restocking data, and nesting) of the Orinoco crocodile, including both published (i.e., scientific articles) and “grey” literature (i.e., technical reports, theses) as well as information provided by experts collected in a previous study [16]. Spatial data (geolocations and localities) from sightings and nests were filtered based on accuracy (i.e., locality described matched with coordinate location) and standardized to the same datum (WGS84) using ArcGIS 10.4 for desktop [20].

We used this spatial information to estimate a species distribution model (SDM) based upon a likelihood analysis for species habitat modeling [21], using a maximum entropy niche analysis (MaxEnt-3.3.3). In this model we included the rarefied occurrence data (see below) and nine different variables: three climate variables (bio 2 -mean diurnal range, bio 4 -temperature seasonality, bio 15 -precipitation seasonality) [22], two physical variables (watershed acc -flow accumulation and dir -flow direction) [23], two eco-physiological variables (global-pet -mean annual potential evapotranspiration and global-aridity -mean annual aridity index) [24], and two landscape variables (lctype -land-cover type and mgvf -maximum green variation factor) [25,26], at ~1 km^2^ resolution. These variables were chosen after testing for spatial autocorrelation (Pearson comparison analysis |r|≤ 0.6) [27] from a pool of 27 variables including all bioclim layers and a digital elevation model.

Graduated spatial rarefying analyses of the occurrence data (rarefied occurrence data) were carried out to eliminate spatial clusters (e.g., over-populated occurrence data over a specific area) and environmental biases [28]. First, we estimated climate heterogeneity of the target area using eigenvalues estimated via principal component analysis (PCA). Based on this analysis, we filtered the occurrence data via multi-distance rarefying analysis using 2 and 25 km as minimum and maximum distances (values defined based on the algorithm) [27].

We selected the background points via buffered local adaptive convex-hull analysis using the maximum radial distance of known occurrence [27]. In this case, we used 10 km as an estimate of the maximum movement per day under average environmental conditions for wild populations. This movement value came from previous work carried out on American crocodiles [2] due to the absence of such an estimate for the Orinoco crocodile. These background points were also compared with the occurrence data to ascertain environmental conditions in which *C*. *intermedius* can potentially occur, avoiding commission errors (false-positives) and over-fitting the model as well as failing to predict *un-colonized climatically suitable habitats* [27,29].

We performed a spatial jackknifing test (geographically structured k-fold cross-validation), dividing the landscape into three regions based on spatial clustering of occurrence points [27]. We also tested five model feature class types (Linear, Quadratic, Hinge, Product, and Threshold), using 5 as a regularization multiplier to optimize the MaxEnt model performance. From these analyses we defined the best model based on the omission rates (the lowest value), the area under the curve (AUC, the highest value) [28], and model feature class complexity (the simplest one) [27]. Finally, we performed a jackknife test of variable importance to define which variable contained the most useful information for the model as well as which one contained information not present in the other variables [27].

We calculated the SDM area removing localities where the species is known to be absent (i.e., zones inhabited by the American crocodile because sympatry for these two species has never been reported) and where geographical barriers impede natural dispersion (i.e., the Andean mountains). We included a 350 m above sea level (asl) isocline as a maximum limit for species distribution because neither historical nor current records have reported this species above this elevation. We determined the *suitable habitat probability threshold* to define the most reliable area in which to analyze habitat characteristics based on the SDM. Using it, we assessed the habitat conservation status based on the most up-to-date land-cover cartography per country [30–32]. Finally, we defined protected and non-protected areas where *C*. *intermedius* populations could potentially inhabit based on the protected areas global database [12].

We temporally analyzed population information by specific locality to define population trends (relative abundance population patterns), as well as to study richness (number of studies per locality) throughout the SDM using *C*. *intermedius* counting methods reported in the literature (i.e., spotlight surveys, daylight surveys, aerial surveys). For this analysis we only used data from localities with at least two nocturnal spotlight surveys [33] carried out in different years to estimate trends. Taking all this information into account (i.e., areas where at least one study has been done), we defined critical areas to preserve/research/monitor and/or generate management actions as RHP/CCU and RRP on the basis of seven ordinal variables classified as discrete values (Table 1). We defined “region” as an area having recognizable characteristics (i.e., Orinoco crocodile populations and studies of this species) but not always fixed boundaries. RHP were defined as areas where the overall conditions are suited to preserve healthy *C*. *intermedius* populations over the long-term that could serve as “refugia” for the species. Areas with high overall values based on this analysis (maximum possible points obtained = 20) were classified as RHP/CCU. Areas with lower overall values were classified as RRP, where the lack of information on *C*. *intermedius* populations prevented a more accurate determination of the species’ conservation status and any direct threats that might reduce the possibilities of hosting populations in the near future. We used a threshold of 60% (≥ 13 points) as the lower limit to define RHP/CCU areas. Areas below this threshold were considered RRP.

**Table 1:** Regional Habitat Priorities/Crocodile Conservation Units (RHP/CCU) and Regional Research Priorities (RRP) variables and classifiers.

Variable	Classifier	Value
Information quality (number of studies and relatedness with the topic -demographic information, habitat conservation status, defined threats)	High (> 5 documents)	3
	Middle (2–5 documents)	2
	Low (< 2 documents)	1
Population status (more than two studies reflecting trends)	Increasing	3
	Stable	2
	Decreasing	1
	Unknown	0
Population size (specific studies including demographic information)	Large/several hundreds	3
	Small/several tens	2
	Very small/few individuals	1
	Unknown	0
Conservation status of the habitat (studies related to land-cover, water conditions, and biodiversity)	Preserved	3
	Low impacted	2
	Highly impacted	1
	Unknown	0
Management actions in place in the area (number of plans and time of action)	Currently active	3
	Historically active	2
	Indirect/inactive	1
	Without actions	0
Protected area	Yes	2
	No	1
Human population in the area (human demographic information and expert consulting)	High (> 500)	1
	Middle (100–500)	2
	Low (< 100)	3

Variables, classifiers, and values used to assess and define RHP/CCU and RRP. Notice the maximum possible value is 20. Classifiers were adjusted based on the number of studies found related to each variable.

## Results

**Fig 1:**
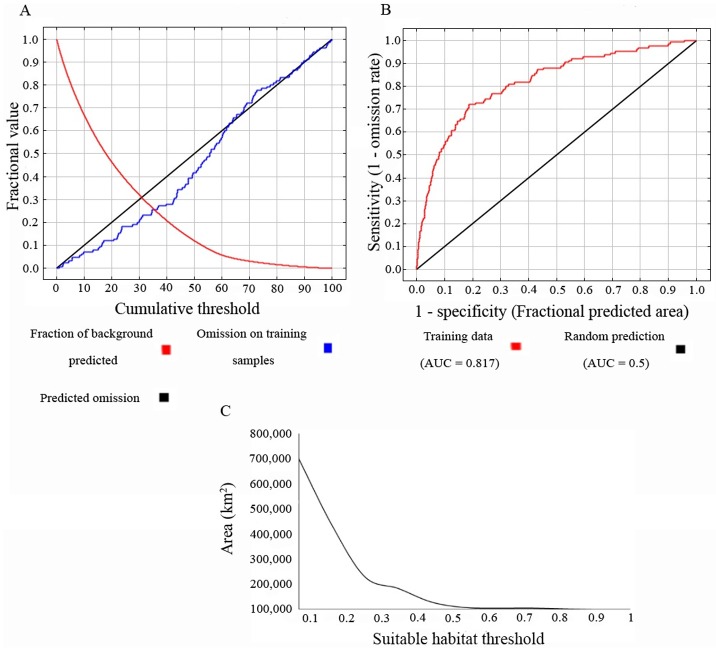
Omission and predicted area, sensitivity vs 1—Specificity, and suitable habitat threshold obtained from the Species Distribution Model (SDM) of the Orinoco crocodile. (A) Omission and predicted area indicating the fraction of background predicted, the predicted omission, and the omission on training samples (B) sensitivity vs fractional predicted area, indicating the area under the curve value (AUC) based on the training data and the random prediction. (C) suitable habitat threshold obtained on the basis of the area covered. Notice the change of the shape of the curve at 0.2 and 0.5 are indicative of breaking points of suitable area cover.

We found and reviewed a total of 289 documents published between 1900 and 2015 dealing with some aspect of the biology or ecology of the Orinoco crocodile in Colombia and/or Venezuela. A total of 1,334 occurrence data points, based on sightings and active nests of *C*. *intermedius*, were collected from reliable sources and used to model, calibrate, and validate the SDM. Of these, 125 geolocations (training samples) were selected after the graduated spatial rarefying analysis was performed. We used a total of 10,090 background points to determine the Maxent distribution, obtaining a regularized and un-regularized training gain of 0.66 and 0.79, respectively. The training value obtained from the area under the curve (AUC) was 0.82; the algorithm reached saturation after 440 iterations (Fig 1).

Based on the results of the jackknife test of variable importance, the environmental variable *watershed acc -flow accumulation* had the highest gain when used in isolation, and therefore appears to contain the most useful information. This same variable decreased the gain the most when omitted and thus appears to contain information that is not present in the other variables.

**Fig 2:**
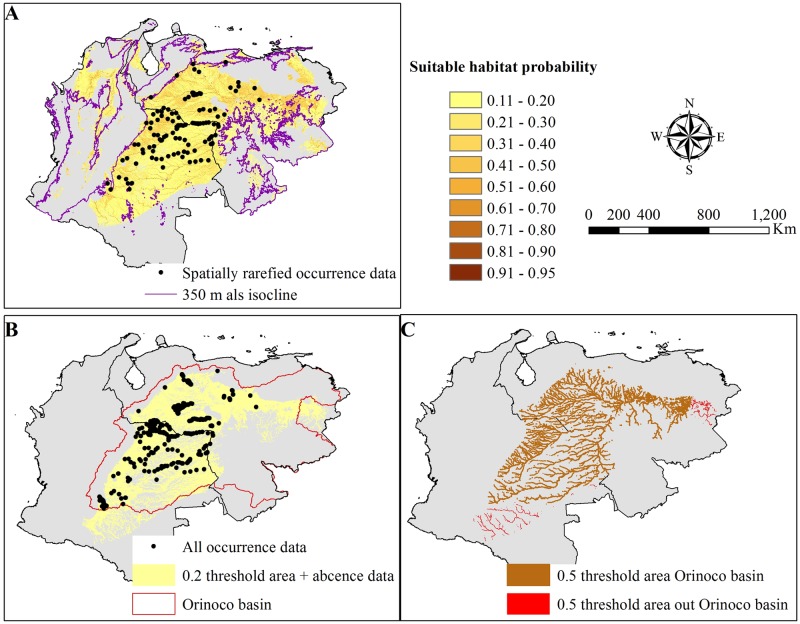
Species Distribution Model (SDM) for the Orinoco crocodile. SDM depicting: (A) all suitable areas available in both Colombia and Venezuela based on the variables assessed as well as the isocline at 350 m asl and the spatially rarefied geolocations; (B) the suitable area including absence data, the 0.2 threshold area, and all the occurrence data used in this study; (C) the most suitable distribution model under the 0.5 threshold area, highlighting areas inside (yellow) and outside (light green) of the Orinoco basin.

We found a change in the slope (threshold) at the 0.2 and 0.5 suitable habitat probability values in the SDM (Fig 1). After removing areas where absence data were available, as well as areas above 350 m asl and areas below the first threshold (<0.2), we obtained a total suitable habitat of 282,277 km^2^ (Fig 2B), of which 3.4% was comprised of in-land waterways (i.e., rivers, creeks, lakes, swamps). Land-cover of the SDM was dominated by grassland in both Colombia and Venezuela (48% of the total area) followed by riparian forest (41.4%) and anthropogenically impacted areas (agricultural and urban zones; 6.7%; Table 2), respectively. Concomitantly, the area obtained with a suitable habitat probability ≥ 0.5 (second threshold) was 23,621 km^2^ (Fig 2C), of which riparian forest represented the highest land-cover type (52%) followed by grassland (27.5%), in-land waterways (13.4%), and anthropogenically impacted areas (6.7%). Of this total area, 10,767 km^2^ are within Colombia and 12.860 km^2^are in Venezuela. Some 2,562 km^2^ are included within protected areas in both Colombia (1,643 km^2^) and Venezuela (919 km^2^), representing only 10.8% of *C*. *intermedius* potential range (Fig 2C).

**Table 2:** Land-cover categories identified on the Orinoco crocodile SDM.

Threshold	0.2				0.5	
Land-cover type	Col. (km^2^)	Ven. (km^2^)	Total	%	Area (km^2^)	%
Riparian forest	65,418	51,490	116,908	41.4	12,288	52.0
Grassland	60,214	75,195	135,409	48.0	6,493	27.5
Waterways	5,124	4,495	9,619	3.4	3,161	13.4
Agriculture	2,072	15,323	17,395	6.2	1,465	6.2
Urban	123	1,358	1,481	0.5	123	0.5
Shrubland	623	811	1,434	0.5	89	0.4
Others	29	2	31	0.0	2	0.0
**Total**	**133,603**	**148,674**	**282,277**		**23,621**	

Land-cover categories identified in the Orinoco crocodile SDM on the basis of a 0.2 and 0.5 threshold scenario. Notice how in the 0.5 scenario the percentage of in-land waterways increased, being more correlated to the amphibious lifestyle of the species.

The SDM included 24 states in both Colombia (10) and Venezuela (14). Of these, Bolívar (4,208 km^2^), Apure (2,609 km^2^), Barinas (1,804 km^2^), Guárico (1,630 km^2^), Portuguesa (709 km^2^), Cojedes (423 km^2^), Anzoátegui (419 km^2^), and Amazonas (391 km^2^) states in Venezuela and Vichada (2,955 km^2^), Casanare (2,200 km^2^), Meta (1,805 km^2^), Guaviare (1,047 km^2^), Guainía (1,008 km^2^), Arauca (856 km^2^), and Caquetá (833 km^2^) departments in Colombia contained the largest suitable habitat for the species based on the variables used in this model. The model also included small areas in states such as Táchira, Vaupés, Cundinamarca, Lara, Aragua, Merida, and Yaracuy (less than 60 km^2^ each). However, these areas can be defined primarily as boundaries or relicts of larger ecosystems present in other states. In the case of the small areas included in the Amazon (Amazonas, Bolívar, Guainía, Caquetá, Guaviare) and Essequibo (Bolívar) basins, it is important to highlight that neither historical nor current records document *C*. *intermedius* in these basins. However, based on the model these areas might be viewed as potential sites for colonization.

**Fig 3:**
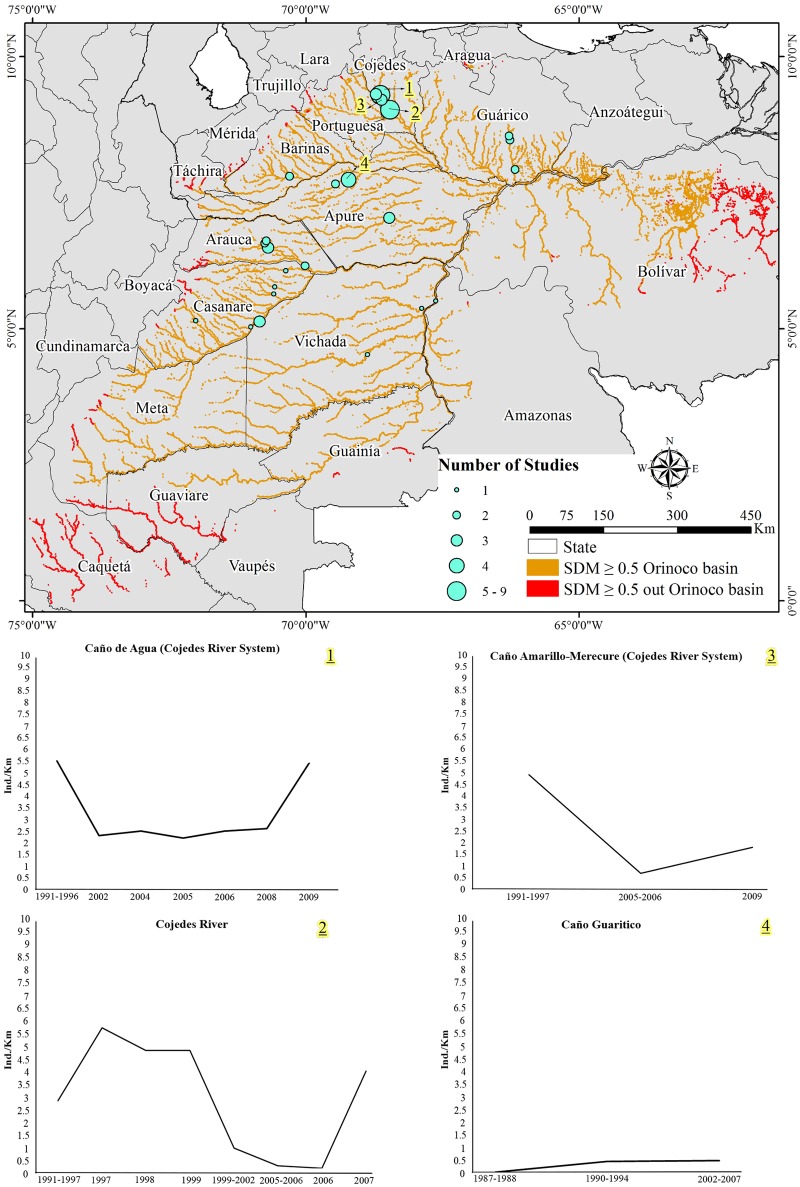
Study richness and relative abundance population patterns. Study richness (number of studies/area) and some relative-abundance population patterns found throughout the Orinoco crocodile range. Notice how research efforts have been localized in just a few areas (e.g., Cojedes River), while others have been essentially neglected (areas with only one study).

We did find 14 localities in Venezuela where population sizes had been estimated using a spotlight survey method (Fig 3, Tables 3 and 4; night time counts determined relative abundance in terms of individual per kilometer -ind/km), with at least two surveys performed through time. These areas, Cojedes River and Caño de Agua (creek), which are both part of the Cojedes River System, were the localities with a greater number of surveys covering a time-span of 10 and 20 years, respectively [9,34,35]. Relative abundance values reported at these 14 localities ranged between 0 ind/km (Anaro River from 1990–1992 and 2000) [36] and 10 ind/km (Laguna de Chigüichigüe-segment of the Manapire River, from 2008–2009) [37]. Overall, crocodile populations increased in relative abundance through time in four of the 14 localities analyzed with Laguna de Chigüichigüe having the highest population growth. In contrast, localities such as Caño Amarillo-Merecure (creek) and Cojedes River reported either a reduction and/or oscillation in relative abundance values recorded during the last 10 years [9] (Fig 3). Furthermore, Caño Guaritico (creek) did not have any reported sightings between 1987 and 1988, but did between 1990 and 1994, 1998 and between 2002 and 2007 [38–40].

**Table 3:** Relative abundances of the Orinoco crocodile populations in Venezuela.

Region	River section	Km	Season	1st survey		2nd survey		≠
				Y.	ind/km	Y.	ind/km	
Capanaparo	Las Campanas	25	Feb	1987	1.12	1989	0.88	-0.24
Capanaparo	Las Campanas	25–26.4	Feb	1989	0.88	2001	1.67	0.79
Capanaparo	Las Campanas	25–23	Jan	1988	1	2011	0.52	-0.48
Capanaparo	Las Campanas	20.4–25	Apr	1986	1.64	2001	1.67	0.03
Capanaparo	Las Campanas	29.3–23	Jun	2001	1.98	2011	0.65	-1.33
Capanaparo	Las Campanas	25–25.4	May	1988	0.56	2001	1.97	1.41
Capanaparo	Naure (N1)	23.5–15	Oct	2000	0.53	2011	0.46	-0.07
Capanaparo	Naure (N2)	14.9–14	Oct	2000	0.6	2011	0.86	0.26
Capanaparo	Piedra Azul (P1)	21–13	Oct	2000	0.24	2011	0.46	0.22
Capanaparo	Piedra Azul (P2)	14.7–14	Oct	2000	0.27	2011	0.21	-0.06
Capanaparo	Piedra Azul (P2)	15–12.6	Jun	2001	0.86	2009	0.56	-0.31
Capanaparo	Piedra Azul (P2)	12.6–14	Jun	2009	0.56	2011	0.5	-0.06
Capanaparo	Piedra Azul (P3)	9.9–14	Oct	2000	0.2	2011	0.5	0.3
Capanaparo	Piedra Azul (P3)	14.9–14	Jun	2001	0.67	2011	0.79	0.11
Cinaruco	Cinaruco	31.4–30	Jun	2001	0	2011	0.1	0.1
Tucupido	Tucupido Reservoir	167–195	Jan-Dec	1991	0.07	2000	0.02	-0.05
Tucupido	Tucupido Reservoir	195–355	Jan-Dec	2000	0.02	2009	0	-0.02
Manapire	Laguna Chigüichigüe	1.7–1.6	Feb	2000	2.35	2009	10	7.65
Manapire	Laguna Larga	2–1.6	Jan-Dec	2000	3.5	2008	6.25	2.75

Comparison of relative abundances of the Orinoco crocodile populations in different regions and sections of rivers in Venezuela including the km surveyed (km) and the times when surveys took place (Y.). Changes are expressed as the differences between the most recent surveys (≠) with respect to the immediately previous one. Some data in this table are the results of averaging surveys from several colleagues [37,47–51].

**Table 4:** Relative abundances of the Orinoco crocodile populations in Venezuela without specific time information.

System	River section	Relative abundance ind/km	Years	Authors
Cojedes River System	Cojedes-Sarare River	3.1	1996–1997	[52–54]
Cojedes River System	Cojedes-Sarare River	2	2005–2006	[52–54]
Cojedes River System	Cojedes-Sarare River	1.93	2006	[52–54]
Apure River	Caño Macanillal (Creek)	1.8	1990–1994	[40,55]
Apure River	Caño Macanillal (Creek)	1.96	2002–2007	[40,55]
Anaro River	Anaro River	0	1990–1992	[56,57]
Anaro River	Anaro River	0	2000	[56,57]

These data are presented in a different table due to the lack of specific information (i.e., collection time and distances surveyed), making direct comparisons impossible. Nevertheless, this information is valuable helping to visualize populations trends.

In Colombia we found 12 localities (Fig 3) where population estimates had been made using a variety of survey methods (i.e., diurnal or nocturnal counts, and/or aerial surveys), of which several did not report the actual distances surveyed (instead they reported number of individuals/sector or zone). Regardless this disparity, “of concern” data (where zero individuals were sighted) were reported from surveys carried out in La Hermosa and Picapico Creeks (1994 and 2010), Ariporo River (1994 and 1995), Cravo Sur River (1995 and 2010), and Tomo River (1995, 1996, and 1997) [41,42]. Other areas such as the Cravo Norte, Casanare, and the Orinoco Rivers, as well as the Guanapalo and Meta River System, have been studied over a 20-year span with a relative abundances ranging from 0.0 to 0.81 ind/km (Casanare River 1996 and 2012; Guanapalo River 2010; Meta River 1995, 1996, 1998, 2010, 2011, 2012; Orinoco River -Vichada sector 1994 and 1995; Cravo Norte River 1994–1995, 2000–2001, 2012) [41–45]. The Lipa and Ele Rivers consistently had a low relative abundance value over a 15-year span (0.2 and 0.3 ind/km in 1995 and 2012; 0.3 ind/km in 1995 and 2001; 0.2 ind/km in 2012) [41,42]. However, colleagues in Arauca [46] reported an increase in populations inhabiting the Lipa, Ele, and Cravo Norte River Systems over a 13 years span based on nest surveys; these increases occurred without any kind of management (wild recovery).

**Fig 4:**
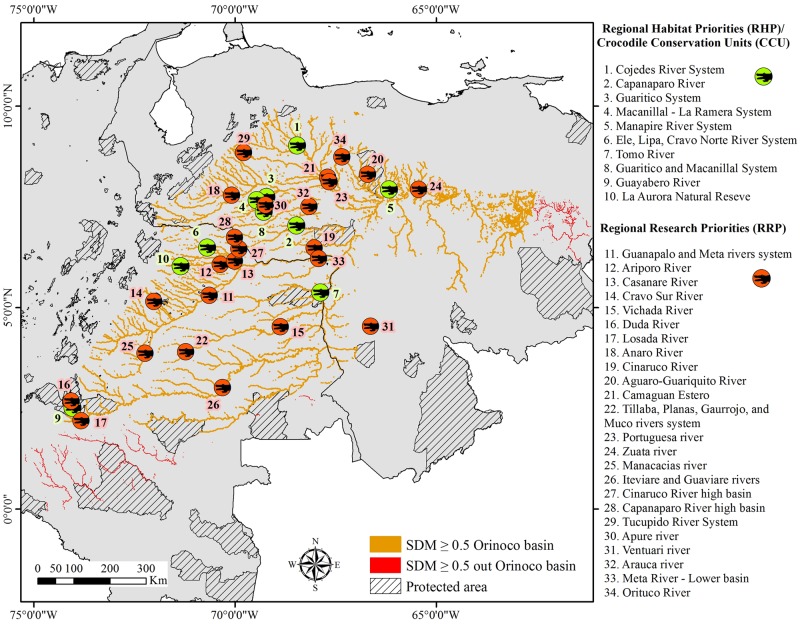
Regional Habitat Priorities/Crocodile Conservation Units (RHP/CCU) and Regional Research Priorities (RRP). RHP/CCU and RRP defined through the Orinoco crocodile range based on current knowledge about the species as well as management actions taking place and relative abundance patterns.

Taking into account all the analyses noted above and based on localities where there are documented reports of *C*. *intermedius* populations, we defined 34 critical areas (16 in Colombia, 17 in Venezuela, and one covering both countries) where we need to preserve/research/monitor and/or generate management actions (Table 5). From these areas, we defined 10 RHP/CCU (six in Venezuela and four in Colombia) and 24 RRP (11 in Venezuela, 12 in Colombia, and one that is shared by both countries; Fig 4). Caño Guaritico (Creek), the Capanaparo River, the Macanillal-La Ramera System, the Cojedes River System, and the Manapire River System in Venezuela were designated as areas with overall conditions (management actions, monitoring and research taking action) conducive to long term preservation of *C*. *intermedius* populations. However, some concerns have arisen regarding the Cojedes River System because its crocodile populations seem to have somewhat declined, even though more than 800 captive-reared individuals have been released there [9,33,34]. On the other hand, the Ele, Lipa, Cravo Norte River System, the Guayabero River, the Tomo River—Tuparro National Natural Park, and the Natural Reserve La Aurora appear to be areas in Colombia with the highest likelihood (in terms of management actions and research) to have maintainable *C*. *intermedius* populations (Table 5). Even though the amount of information from these populations is not as complete as from those areas described in Venezuela, these systems seem to represent the most suitable areas in which to maintain healthy populations of this species.

**Table 5:** Regional Habitat Priorities/Crocodile Conservation Units (RHP/CCU) and Regional Research Priorities (RRP).

Co	Department/State	Area defined	IQ	PSt	PSi	HS	MA	PA	HP	Sc
Ven	Apure	Guaritico System (Caño Guaritico)	3	3	2	3	3	2	3	19
Ven	Apure	Capanaparo River	3	1	3	3	3	2	3	18
Ven	Apure	Macanillal-La Ramera System	3	3	3	1	3	2	3	18
Col	Arauca	Ele, Lipa, Cravo Norte River System	3	3	3	3	2	1	2	17
Ven	Cojedes	Cojedes River System (Caños de Agua and Amarillo, Cojedes-Sarare and Cojedes Rivers)	3	1	3	1	3	1	2	14
Col	Casanare	La Aurora Natural Reserve	2	0	1	3	3	3	2	14
Ven	Guárico	Manapire River System (Lagunas Larga and Chigüichigüe and Manapire River)	2	1	1	2	3	1	3	13
Ven	Apure	Guaritico and Macanillal System (El Cedral Ranch, Matiyure, and Caicara creeks)	2	1	1	2	3	1	3	13
Col	Meta	Guayabero River	2	0	2	2	3	2	2	13
Col	Vichada	Tomo River—(Tuparro National Natural Park)	1	0	1	3	3	2	3	13
Col	Meta	Duda River	2	0	2	3	0	2	3	12
Col	Meta	Losada River	1	0	2	2	3	2	2	12
Ven	Barinas	Anaro River	1	1	1	3	2	1	2	11
Ven	Apure	Cinaruco River	1	1	1	2	1	2	3	11
Col/Ven	Vichada	Meta River—Lower basin	2	1	1	3	1	1	2	11
Ven	Guárico	Aguaro-Guariquito River	1	0	1	2	1	2	3	10
Ven	Guárico	Camaguan Estero	1	0	1	2	2	2	2	10
Col	Vichada	Tillava, Planas, Guarrojo, and Muco Rivers System	1	0	1	3	0	1	3	9
Col	Vichada	Vichada River	1	0	1	3	0	1	3	9
Ven	Portuguesa	Portuguesa River	1	0	1	3	0	1	3	9
Ven	Guarico	Zuata River	1	0	1	3	0	1	3	9
Col	Meta	Manacacias River	1	0	1	2	0	1	3	8
Col	Guavire/Vichada	Iteviare and Guaviare Rivers	1	0	0	3	0	1	3	8
Col	Arauca	Cinaruco River high basing	1	0	0	3	0	1	3	8
Col	Arauca	Capanaparo River	1	0	0	3	0	1	3	8
Ven	Portuguesa	Tucupido River System	1	0	1	1	2	1	2	8
Ven	Apure/Barinas	Apure River	1	0	1	3	0	1	2	8
Ven	Amazonas	Ventuari River	1	0	1	2	0	1	3	8
Ven	Apure	Arauca River	1	0	1	3	0	1	2	8
Col	Casanare	Ariporo River	1	0	0	1	2	1	2	7
Col	Meta/Casanare	Guanapalo and Meta Rivers System (La Hermosa and Picapico creeks)	1	0	0	2	0	1	3	7
Ven	Guárico	Orituco River	1	0	1	0	2	1	2	7
Col	Casanare	Cravo Sur River	1	0	0	2	0	1	2	6
Col	Casanare	Casanare River	1	0	0	2	0	1	1	5

Orinoco crocodile RHP/CCU (≥ 13 points in score) and RRP (< 13 points score) defined based on historical and current studies carried out throughout its range. Country (Co), Colombia (Col), Venezuela (Ven), Information Quality (IQ), Population Status (PSt), Population Size (PSi), Conservation status of the habitat (HS), Management actions in place in the area (MA), Protected area (PA), Human population in the area (HP), Score (Sc). Ranks per variable oscillate between 0 and 3 with the exception of IQ (1 to 3) and PA (1 and 2).

Areas such as the Casanare, Cravo Sur, Meta, Ariporo, Cinaruco, Iteviare, and Guaviare Rivers in Colombia and the Orituco, Arauca, Ventuari, Apure, Tucupido, Zuata and Portuguesa Rivers in Venezuela were chosen as RRP because *C*. *intermedius* has historically been studied in these areas. unfortunately, we do not have enough reliable information to accurately infer population numbers and/or determine the status of the species; furthermore, currently no formal management actions are taking place (Table 5).

## Discussion

This study is one of the very few comprehensive analyses of Orinoco crocodile biology, which includes all available ecological information as well as develops an SDM to assess its conservation status and identify RHP/CCU and RRP. To date only one other SDM analysis has been done for the Orinoco crocodile that focused on areas determined to be suitable for this species [16,58]. However, they failed to predict thresholds (representativeness of the model), areas (no area estimation) and variables of influence due to the lack of spatial autocorrelation analyses and graduated spatial rarefying analyses of the occurrence data [27]. Thus, this study is the first to attempt to estimate suitable habitat of the Orinoco crocodile (and one of the first for any crocodylian) under the conditions defined in this analysis (23,621 km^2^), with Venezuela supporting the larger proportion of the species range (12,860 km^2^) compared to Colombia (10,767 km^2^). While these data should not be viewed as absolute values, they do provide the first approximation to quantify the range of the Orinoco crocodile under a robust analysis. Our results also suggest that this type of research must continue in order to collect more spatial information, which will allow researchers to improve the present model.

Niche modelling represents a valuable strategy for estimating suitable potential habitat for species, but it should be done with a clear understanding of the assumptions and predictions resulting from the model [27]. The prediction of a suitable area under certain variables needs to be analyzed in the context of the species’ ecology and how accurate the model is describing it (proportion values). Graphical tools can help researchers define break points (Fig 1) in the distribution of the data, which can provide information about how representative the model is and under what proportion value area analyses should be done. In this study, we found two break points in the distribution of the data derived from the model (at 0.2 and 0.5). The 0.5 proportion value indicated a larger number of in-land waterways (i.e., rivers, creeks, lakes, swamps), which accurately reflects the basic ecology of the species [8,40]. Riparian forest and grasslands are the most common types of landscape occupied by Orinoco crocodiles, reflecting considerable more optimal habitat as compared to anthropogenic areas. Watershed acc- flow accumulation was the most influential environmental variable in this model, probably because it reflects catchment water areas (i.e., rivers, creeks, and swamps), which are the main habitats where *C*. *intermedius* occurs [8]; this finding makes watershed the most relevant variable to define the SDM in this study.

Although the Orinoco crocodile is primarily restricted to aquatic systems (recognizing that these are composed of both water and beach), the habitat analysis shows a larger amount of land-cover than in-land water. This could be due to a bias in the cartography used to analyze it [30–32], since the standardized method used to create land-cover layers focused mainly on obtaining land-cover areas and neglecting linear water bodies (i.e., rivers, creeks, streams) below a certain width [30]. Therefore, a method of remote sensing interpretation that focuses specifically on in-land waters must be developed to improve the quality of habitat analysis of semiaquatic species such as the Orinoco crocodile.

The SDM identified several areas outside of the Orinoco watershed (Fig 2C) in the departments of Caquetá, Southern Guaviare, Vaupes and Southern Guainía in Colombia and the states of Bolívar and Amazonas in Venezuela. Some of these areas report sporadic (or historical) presence of the species (Guaviare Department, Inirida River, Ventuari River, and Caura River) [8,59,60], and others have had no research on crocodiles that could reject this hypothesis. Nevertheless, based on the variables included in the SDM, these small areas (which make up part of the hydrographic boundaries between the Orinoco and Amazon basins) might be treated as suitable habitats for potential colonization by *C*. *intermedius*. This has important implications for the conservation of this species because species ranges are not static, but are dynamic and range-shifting has been documented in several species due to anthropogenic or climatic pressures [61]. However, to date no modelling that incorporated climate change effects on crocodylians has been done, which creates a gap in our understanding about how these species might respond to changes in climate.

One feature generally described as the main driver of the distribution and number of Orinoco crocodiles found at a particular locality is productivity (i.e., both primary and secondary) of water bodies, commonly defined in the Orinoco basin as white water (high productivity) clear water (medium productivity) and black water (low productivity) [7,16,62,63]. Attempts have been made in the Orinoco basin to map these attributes based on major rivers and a categorical classification [16]. However, continuous productivity water layers must be created based on primary and secondary productivity estimates of water bodies to spatially test how these variables might affect the range of the species (expanding or shrinking the range estimated in the present model).

The Orinoco savanna has been identified as a high priority ecoregion for biodiversity conservation due to its habitat singularity and uniqueness in the world [64]. This area is currently catalogued as “vulnerable” due to landscape transformation [65]. An important component of this ecosystem is the Orinoco crocodile, which has been historically described as restricted to this habitat [8]. Despite these apparent attributes, only 10.8% of the suitable habitat estimated for *C*. *intermedius* within the Orinoco savanna is protected (either as a natural park or wildlife refuge) in either Colombia and Venezuela. Although Venezuela contains a larger area of savanna (68% of the entire savanna ecoregion) compared to Colombia, the amount of potentially protected range in this country is smaller (919 km^2^ as compared to 1,643 km^2^ in Colombia). It is important to highlight that Venezuela has two national parks within the Orinoco crocodile’s range, one of them (Cinaruco-Capanaparo) whose borders were modified in 1992 to include one of the most important populations of the species [66]. There is also a wildlife refuge (Caño Guaritico), established in 1989 with the very specific aim of protecting this species [67]. These two protected areas have received more than 48% of captively-reared *C*. *intermedius* released into the wild in Venezuela since 1990 [68].

Regarding this last point, Venezuela has done an outstanding job restocking and recovering *C*. *intermedius* populations over the past three decades [68]; this certainly has influenced the population dynamics patterns depicted in the present analyses. To date, 9,812 individuals have been released in consecutive years between 1990 and 2016 in five states (Apure, Guárico, Barinas, Cojedes, and Portuguesa), with a maximum of 763 individuals released during 2009 and a minimum of 40 during 1990 [68,69]. In contrast, even though efforts at restocking in Colombia have not been as vigorous as in Venezuela, it is notable that starting in 2015 two institutions (The Palmarito Foundation and the National University of Colombia) have released a total of 57 individuals in three departments (Casanare, Vichada, and Meta) [70–73].

One of the most important achievements in Venezuela in terms of recovery of *C*. *intermedius* is the establishment of a population in the El Frio biological station—Caño Guaritico (creek) and surrounding areas in Apure state [40,74]. However, a number of authors are concerned about the long-term viability of this population because of habitat transformation and anthropogenic pressures (mainly illegal hunting) [67]. In Colombia, the assessment that is currently being carried out in Meta and Vichada departments will hopefully more accurately determine home ranges, utilization distributions, and movement patterns of different age groups using different methodologies (GPS and VHF tags) [69,72], and will provide useful information for planning and management actions. However, additional efforts will have to put in place to ensure the viability of C. *intermedius*, both increasing the number of captive breeding and reintroduction programs and allowing the largely diminished wild populations in Colombia to recover.

Historically, four areas in Venezuela have been monitored over the long-term: The Cojedes River System [9,34,35,52,75], the Capanaparo River [47–49,76,77], the Manapire River [37,51,62], and the Caño Macanillal-Laguna la Ramera [38,40]. However, we can now estimate relative abundance patterns from 14 areas, allowing at least a preliminary assessment of the population demographics in Venezuela (Fig 3, Tables 3 and 4). Laguna de Chigüichigüe had the highest relative abundance value reported to this point for the species (10 ind/km). Overall, five out of the 14 localities analyzed increased in relative abundance, six had a reduction and three appear to be relatively stable through time (Fig 3, Tables 3 and 4). However, the surveyed distances in some of these localities are relatively short, so the reported relative abundance values must be taken with caution. One area “of concern” is the Tucupido River where *C*. *intermedius* populations have been impacted due to construction of a reservoir in 1988. Since then, populations in this area have been declining sharply and no individuals were seen during the last survey, which was conducted in 2009 [62]. Therefore, conservation measures must be put in place immediately to accurately assess and hopefully refurbish populations in this area.

In Colombia, we did find relative abundance patterns for some areas such as La Hermosa and Picapico creeks as well as in the Ariporo, Cravo Sur, and Tomo Rivers (zero individuals sighted) [41,42]. However, the variety of methods used to estimate population attributes make it difficult to clearly define whether these tendencies are artificial (due to the lack of uniformity). It is also difficult and complicated to make comparisons between countries regarding the overall conservation status of this species. However, areas such as the Ele, Lipa, Cravo Norte River System have documented increased numbers of *C*. *intermedius* over the last two decades, so efforts should be made to define the current status of the species. Efforts in the Guayabero River should be increased to better assess population status and accurately estimate population size, neither of which at present is clear.

It is important to note that while the relative abundance patterns reported in our study may accurately reflect the current state of knowledge for the Orinoco crocodile, the lack of continuous monitoring in several areas such as the Manapire River (Guarico state), Anaro River (Barinas state), and Caño Guaritico (Apure state), as well as in the Ele, Lipa, Cravo Norte River System in Colombia, may have biased these abundance patterns. Nest counts in the Ele, Lipa, Cravo Norte River System [46] showed a population increase much higher than the data reported by spotlight surveys data [41–44]. However, the authors of the first study highlight the possibility of bias due to sampling effort. These issues emphasize the necessity to define a standardized protocol (i.e., nocturnal spotlight surveys and nest counts in a defined number of localities at the same time period each year) for researchers and government authorities working on *C*. *intermedius* to collect both robust and comparable information. Such coordinated approaches will provide greater clarity in determining population tendencies, generating useful information for the development of optimal management actions and planning [5]. It is notable that when comparing the two countries efforts to recover *C*. *intermedius* populations, Colombia is nowhere close to Venezuela in terms of management actions currently in place. Therefore, “catching-up” should be a high priority to preserve the Orinoco crocodile in Colombia.

The Cojedes River System is one of the most well-known areas and is considered to harbor the most important *C*. *intermedius* population in its entire range [9,52,75]. However, up to this point potential adjacent-habitats (i.e., several rivers and creeks) have been neglected with all research effort focused on the southern end of Caño de Agua and the Merecure-Caño Amarillo section of the Cojedes River, apparently due to logistical constraints [9]. Nevertheless, this area along with Capanaparo River and several river systems including the Guaritico, the Macanillal-La Ramera, and the Manapire in Venezuela and the Ele, Lipa, Cravo Norte River System and Guayabero River in Colombia (Table 5) are the RHP/CCU that seem to be most well suited for long-term preservation of *C*. *intermedius* populations. Even though some management adjustments will be necessary (primarily in the Colombian areas) to increase the research effort, these RHP/CCU can be used as *in vivo* laboratories to define important ecological attributes of the species that will provide the additional knowledge necessary to preserve Orinoco crocodiles. Areas defined as RRP such as the Casanare River, the Cravo Sur, and Meta River System and the Tucupido River System (Table 5) will require strong efforts in the near future to establish monitoring strategies that can accurately assess the conservation status of the Orinoco crocodile and develop management actions to preserve them.

The RHP/CCU and RRP areas defined in the current study are not meant to be definitive, but rather to present a baseline that can help to formulate efficient and sound conservation strategies. This study should be used as a first step in a coordinated effort to build a comprehensive data set through time. Furthermore, it must be remembered that SDM, RHP, and RRP are all dynamic depending upon the level of understanding we have about populations and the pressures (stochastic or anthropogenic) they are under. Recommendations based on the current study do not pretend to be novel as management plans developed for Orinoco crocodiles over the last four decades have stated similar necessities to recover the species [15–19]. Instead, the current study highlight in detail what we have done and what we feel needs to be done to preserve the Orinoco crocodile based on regional priorities. It has been clear for the last 40 years that not all regions within the two countries where the species ranges have had the same needs or priorities, thus redefining *regional* priorities will create a route that can be followed in a cost-effective manner.

Overall, only 10 out of the 34 areas we assessed have the necessary conditions to support the species over the long-term (RHP/CCU), which is always “of concern” when talking about a threatened taxon. Even though local conservation strategies have had some successes across the range of the Orinoco crocodile, the overall conservation status of the species is still critical; this reemphasizes the need to increase the efforts to recover it (especially in Colombia) to ensure its survival as a functional and important part of the ecosystems it inhabits. We are aware that continuous monitoring of 34 areas is logistically and financially unbearable in countries such as Colombia and Venezuela where the primary concern for governments is often economic development rather than the protection of natural resources. Nevertheless, a consensus among all the players with a vested interest in the preservation of this species (e.g., NGO’s, governmental entities, academic units, and researchers) must be a priority and must be based on a plan where both the benefit and cost are acceptable to all parties.
